# A survey of hormone replacement therapy use among resistance-trained women across states of menopause transition – a preliminary analysis

**DOI:** 10.1080/15502783.2025.2550214

**Published:** 2025-08-26

**Authors:** John Holtje, Anna Oelmann, Valeria V. Silva Ramirez, Gretchen Shelton, Landon Shannahan, Nam Nguyen, Jacey Greece, Laura Pyott, Stacy Ann Cunnington, Laurin Conlin, Vonda J. Wright, Bill I. Campbell

**Affiliations:** a University of South Florida, Performance & Physique Enhancement Lab, Exercise Science & Kinesiology Program, Tampa, FL, USA; b Boston University School of Public Health, Department of Community Health Sciences, Boston, MA, USA; c West Chester University, Department of Mathematics, West Chester, PA, USA; d University of Central Florida, College of Medicine, Orlando, FL, USA

**Keywords:** Females, menopause transition, health, hormone replacement

## Abstract

**Background:**

Hormone Replacement Therapy (HRT) is considered the gold standard for management of vasomotor symptoms. It can also help improve bone health, potentially reducing the risk of osteoporosis and fractures. There is current debate regarding the use of HRT and its risk-to-benefit ratio for cardiovascular and cognitive health. While there is a plethora of research reporting HRT use among the general population, research on HRT use among resistance-trained women is lacking. In the United States, HRT use among the general population of post-menopausal women has rapidly declined. It was estimated to be 4.7% in 2020, down from 26.9% in 1999. The purpose of this study was to survey HRT usage among post-menopausal resistance-trained women.

**Methods:**

The present study is part of a larger overall female fitness menopause survey consisting of 132 questions that were developed collaboratively by the authors and conducted through Qualtrics Software (Qualtrics, Provo, UT, USA). The survey was distributed via online platforms and relied on voluntary participation, which may have limited the respondent pool to individuals with internet access and higher digital literacy. This preliminary descriptive analysis focused on the question: “Are you currently using HRT?” with three close-ended responses: “Yes”; “No”; “I prefer not to answer.” In the survey, HRT use was defined as taking estrogen or progesterone. Data is presented descriptively.

**Results:**

A total of 943 subjects identified themselves as post-menopausal. Of these, 514 reported to be currently using HRT (55% of total respondents).

**Conclusion:**

This study suggests, within the population of self-identified post-menopausal resistance-trained women, there may be a much larger rate of HRT usage than previously reported. One notable limitation of this study is the use of a survey disseminated through social media platforms. This approach introduces potential selection bias, as users of social media platforms may not reflect the broader population in terms of socioeconomic status, education, and other demographic or behavioral characteristics. Additionally, the voluntary nature of survey participation on social media may lead to self-selection bias, whereby individuals with strong opinions or vested interest in the topic are more likely to respond. As such, the sample may over-represent particular demographic groups or viewpoints, limiting the generalizability of the findings. Future research should consider using stratified sampling or supplementing online recruitment with offline methods to ensure broader representation.

